# Intranasal plus subcutaneous prime vaccination with a dual antigen COVID-19 vaccine elicits T-cell and antibody responses in mice

**DOI:** 10.1038/s41598-021-94364-5

**Published:** 2021-07-21

**Authors:** Adrian Rice, Mohit Verma, Annie Shin, Lise Zakin, Peter Sieling, Shiho Tanaka, Joseph Balint, Kyle Dinkins, Helty Adisetiyo, Brett Morimoto, Wendy Higashide, C. Anders Olson, Shivani Mody, Patricia Spilman, Elizabeth Gabitzsch, Jeffrey T. Safrit, Shahrooz Rabizadeh, Kayvan Niazi, Patrick Soon-Shiong

**Affiliations:** ImmunityBio, Inc., 9920 Jefferson Blvd, Culver City, CA 90232 USA

**Keywords:** Drug discovery, Immunology

## Abstract

We have developed a COVID-19 vaccine, hAd5 S-Fusion + N-ETSD, that expresses SARS-CoV-2 spike (S) and nucleocapsid (N) proteins with modifications to increase immune responses delivered using a human adenovirus serotype 5 (hAd5) platform. Here, we demonstrate subcutaneous (SC) prime and SC boost vaccination of CD-1 mice with this dual-antigen vaccine elicits T-helper cell 1 (Th1) biased T-cell and humoral responses to both S and N that are greater than those seen with hAd5 S wild type delivering only unmodified S. We then compared SC to intranasal (IN) prime vaccination with SC or IN boosts and show that an IN prime with an IN boost is as effective at generating Th1 biased humoral responses as the other combinations tested, but an SC prime with an IN or SC boost elicits greater T cell responses. Finally, we used a combined SC plus IN (SC + IN) prime with or without a boost and found the SC + IN prime alone to be as effective in generating humoral and T-cell responses as the SC + IN prime with a boost. The finding that SC + IN prime-only delivery has the potential to provide broad immunity—including mucosal immunity—against SARS-CoV-2 supports further testing of this vaccine and delivery approach in animal models of viral challenge.

## Introduction

In response to the need for a COVID-19 vaccine that is safe, effective, and suitable for global distribution, we have developed the dual antigen hAd5 S-Fusion + N-ETSD vaccine including formulations for subcutaneous (SC), oral, and intranasal (IN) delivery. The vaccine comprises the SARS-CoV-2 spike (S) protein modified for enhanced cell surface expression (S-Fusion) to increase humoral responses and the nucleocapsid (N) protein with an Enhanced T-cell Stimulation Domain (N-ETSD) to target N to the endosomal/lysosomal cellular compartment^1^ to enhance MHC class I and II presentation.

The vaccine antigens are delivered using a human adenovirus serotype 5 (hAd5) vector with deletions in the E1, E2b, and E3 gene regions (hAd5 [E1-, E2b-, E3-])^2^. Removal of E2b gene regions results in a reduction of late gene expression of viral protein such as the Ad5 viral fiber protein and allows for expression of inserted transgenes for extended periods of time even in the presence of pre-existing Ad5 immunity^3–7^. The platform therefore shows potential to be suitable for homologous prime-boost immunization and/or immunotherapy regimens^8–12^. Importantly, this next generation Ad vector has demonstrated safety in over 125 patients with solid tumors. In these Phase I/II studies in cancer patients, CD4 + and CD8 + antigen-specific T cells were successfully generated to multiple somatic antigens (CEA, brachyury, MUC1, PSA) even in the presence of pre-existing Ad immunity^8,11,12^.

SARS-CoV-2 is an enveloped positive sense, single-strand RNA β coronavirus primarily composed of four structural proteins—S, N, membrane (M), and envelope (E)—as well as the viral membrane and genomic RNA. The S glycoprotein^13–15^ is displayed as a trimer on the viral surface, whereas N is located within the viral particle. Spike initiates infection by the SARS-CoV-2 virus by interaction of its receptor binding domain (RBD) with human host angiotensin-converting enzyme 2 (ACE2) expressed on the surface of cells in the respiratory system, including alveolar epithelial cells^16^, as well as cells in the digestive tract.

The majority of current SARS-CoV-2 vaccines under development deliver only the S antigen because antibodies raised against S RBD are expected to neutralize infection^17–19^. Reliance on S as the sole vaccine antigen is not without risk, however, particularly in the face of the rapidly dominating variants including the B.1.351 variant expressing E484K, K417N, and N501Y mutations^20^; the B.1.1.7 variant (N501Y)^21,22^; and the Cal.20C L452R variant^23^ all of which have altered RBD sequences that may not be as effectively recognized by antibodies generated in response to first-wave sequence S-based vaccines^24–26^.

To lessen the risk of single-antigen delivery and to broaden protective immune responses, we included the N protein in our hAd5 S-Fusion + N-ETSD vaccine. N is a highly conserved and antigenic SARS-CoV-2-associated protein that has been studied previously as an antigen in coronavirus vaccine design for SARS-CoV^27–30^. N associates with viral RNA and has a role in viral RNA replication, virus particle assembly, and release^31,32^. Studies have shown that nearly all patients infected with SARS-CoV-2 have antibody responses to N^33,34^. Furthermore, another study reported that most, if not all, COVID-19 survivors tested were shown to have N-specific CD4 + T-cell responses^19^.

The ability of N to elicit vigorous T-cell responses highlights another advantage of the addition of N. A robust T-cell response to vaccination is at least as important as the production of antibodies^35^ and should be a critical consideration for COVID-19 vaccine efficacy. First, humoral and T-cell responses are highly correlated, with titers of neutralizing antibodies being proportional to T-cell levels, suggesting the T-cell response is necessary for an effective humoral response^36^. It is well established that the activation of CD4 + T helper cells enhances B-cell production of antibodies. Second, virus-specific CD4 + and CD8 + T cells are widely detected in COVID-19 patients^37^, based on findings from patients recovered from the closely-related SARS-CoV, and there are reports that such T cells persist for at least 6–17 years, suggesting that T cells may be an important part of long-term immunity^38–40^. These T-cell responses were predominantly to N, as described in Le Bert et al*.*, who found that in all 36 convalescent COVID-19 patients in their study, the presence of CD4 + and CD8 + T cells recognizing multiple regions of the N protein could be demonstrated^40^. They further examined blood from 23 individuals who had recovered from SARS-CoV and found that the memory T cells acquired 17 years ago also recognized multiple proteins of SARS-CoV-2. These findings emphasize the importance of designing a vaccine with the highly conserved N present in both SARS-CoV and SARS-CoV-2. Third, recovered patients exposed to SARS-CoV-2 have been found without seroconversion, but with evidence of T-cell responses^41^. T-cell based responses become even more critical given the finding in at least one study that neutralizing antibody titers decline in some COVID-19 patients after about 3 months^42^. The importance of both S and N was highlighted by Grifoni et al.^19^ who identified both antigens as a priori potential epitopes that are predicted to induce both B and T cell responses to the SARS-CoV virus that is similar to SARS-CoV-2.

While we find the evidence that cell-mediated protection is a key element for vaccine efficacy, we note its role, and specifically the contribution of N-elicited T-cell responses, to protection against SARS-CoV-2 infection has not been established in animals models.

Additional considerations for vaccine design beyond the choice of antigens include the ability to generate mucosal immunity that provides the highest probability of preventing transmission. IN delivery offers the potential to confer mucosal immunity. SARS-CoV-2 is a mucosal virus^43, 44^ that in most instances, initiates infection by entry to the nose and mouth. Similarly, it’s most efficient route of transmission is by respiratory droplets that are then transmitted to other persons^45^. Thus a vaccine that also elicits protective mucosal responses mediated by IgA is more likely to reduce transmission as compared to systemic, IgG-only humoral and T-cell responses^46^.

It was our goal in the studies presented herein to confirm enhanced cell surface expression of S-Fusion as compared to S-wild type (S-WT) in in vitro studies, then compare humoral and T cell responses after vaccination with either hAd5 S-WT or S-Fusion + N-ETSD in in vivo studies in CD-1 mice. Here, after we established generation of anti-S antibodies and S-reactive T-cell responses were greater with hAd5 S-Fusion + N-ETSD than hAd5 S-WT, we then compared SC and IN prime delivery of the hAd5 S-Fusion + N-ETSD vaccine. In a third experiment, the two routes of delivery were combined in a single prime to ascertain if together optimal immune responses could be achieved that may not necessarily be dependent upon a boost.

In all three study paradigms—SC prime with SC boost study, SC versus IN prime with boost, and combined SC plus IN prime with or without boost—immunization of CD-1 mice with the hAd5 S Fusion + N-ETSD vaccine elicited Th1 biased humoral responses against S. Both CD4 + and CD8 + T-cell responses to SARS-CoV-2 S and N peptide pools were also seen, with cytokine production being greater overall in response to N peptides. Potent neutralization of SARS-CoV-2 by sera from vaccinated mice in all studies was confirmed by a surrogate neutralization assay^47^. While all dosing paradigms produced broad immune responses, perhaps the most significant and compelling finding was that a single prime administration by combined SC and IN dosing generated immune responses that were at least as great as dosing regimens that included a boost.

## Results

### hAd5 S-Fusion and hAd5 S-Fusion + N-ETSD show enhanced cell-surface display of conformationally-relevant S RBD as compared to S-WT

**Figure 1:**
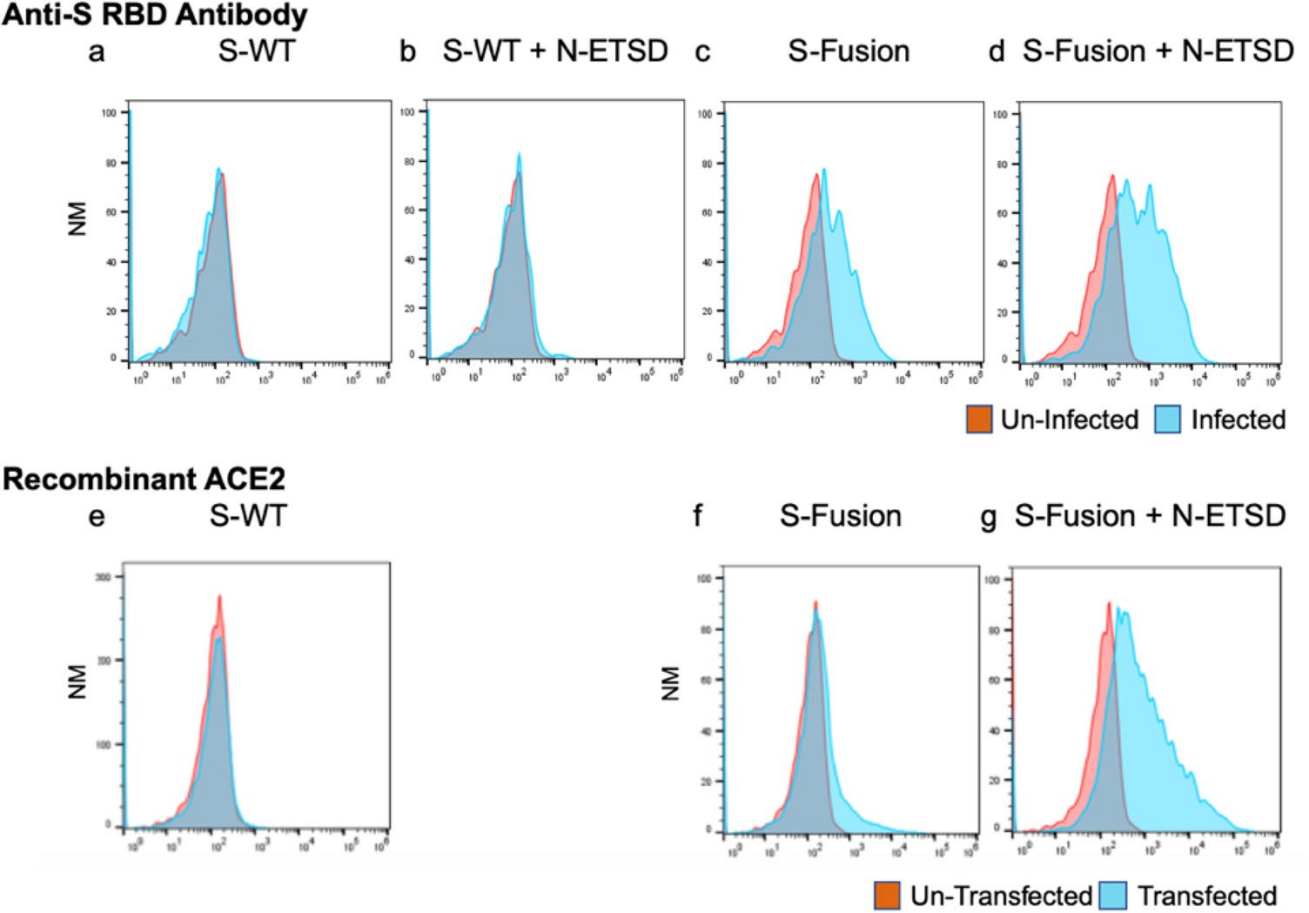
*HEK-293T cells expressing hAd5 S-Fusion* + *ETSD show enhanced spike receptor binding domain (S RBD)-specific antibody and recombinant ACE2 (rACE2) binding.* Flow cytometric analysis of anti-S RBD antibody binding to construct-infected cells reveals surface expression of S RBD is very low in (**a**) S-WT or (**b**) S-WT + N-ETSD infected cells and is higher in (**c**) S-Fusion infected cells. The highest S RBD cell surface expression was seen for (**d**) S-Fusion + N-ETSD infected cells. rACE2 showed little binding to HEK-293T cells transfected with (**e**) S-WT, higher binding with (**f**) S-Fusion, and the highest binding with (**g**) S-Fusion + N-ETSD. Y-axis scale is normalized to mode (NM).

Before initiation of in vivo studies in mice, our goal of enhancing cell-surface display of S was confirmed by transduction (infection) of HEK-293T cells with hAd5 S-WT, S-WT + N-ETSD, S-Fusion alone, and S-Fusion + N-ETSD followed by flow cytometric analysis of anti-S RBD antibody binding. As shown in Fig. 1, there was very little binding of anti-S RBD-specific antibodies to the surface of HEK 293T cells transduced with hAd5 S-WT (Fig. 1a) or hAd5 S-WT + N-ETSD (Fig. 1b) constructs; antibody binding to hAd5 S-Fusion was higher (Fig. 1c). The highest cell-surface expression of RBD was detected after transduction with dual antigen hAd5 S-Fusion + N-ETSD (Fig. 1d).

Similar results were seen for recombinant angiotensin converting-enzyme 2 (ACE2)-Fc binding to HEK 293T cells transfected with hAd5 S-WT, S-Fusion or S-Fusion + N-ETSD; with ACE2 showing higher binding to S-Fusion than S-WT and the dual antigen construct showing the highest binding (Fig. 1e–g).

These findings support our rationale for modification of S with the fusion sequence that was predicted to increase cell-surface display of physiologically-relevant S.

### The hAd5 S-WT versus hAd5 S-Fusion + N-ETSD SC prime and boost study in CD-1 mice

#### SC prime and boost vaccination with hAd5 S-Fusion + N-ETSD elicits higher anti-S IgG generation than hAd5 S-WT

**Figure 2:**
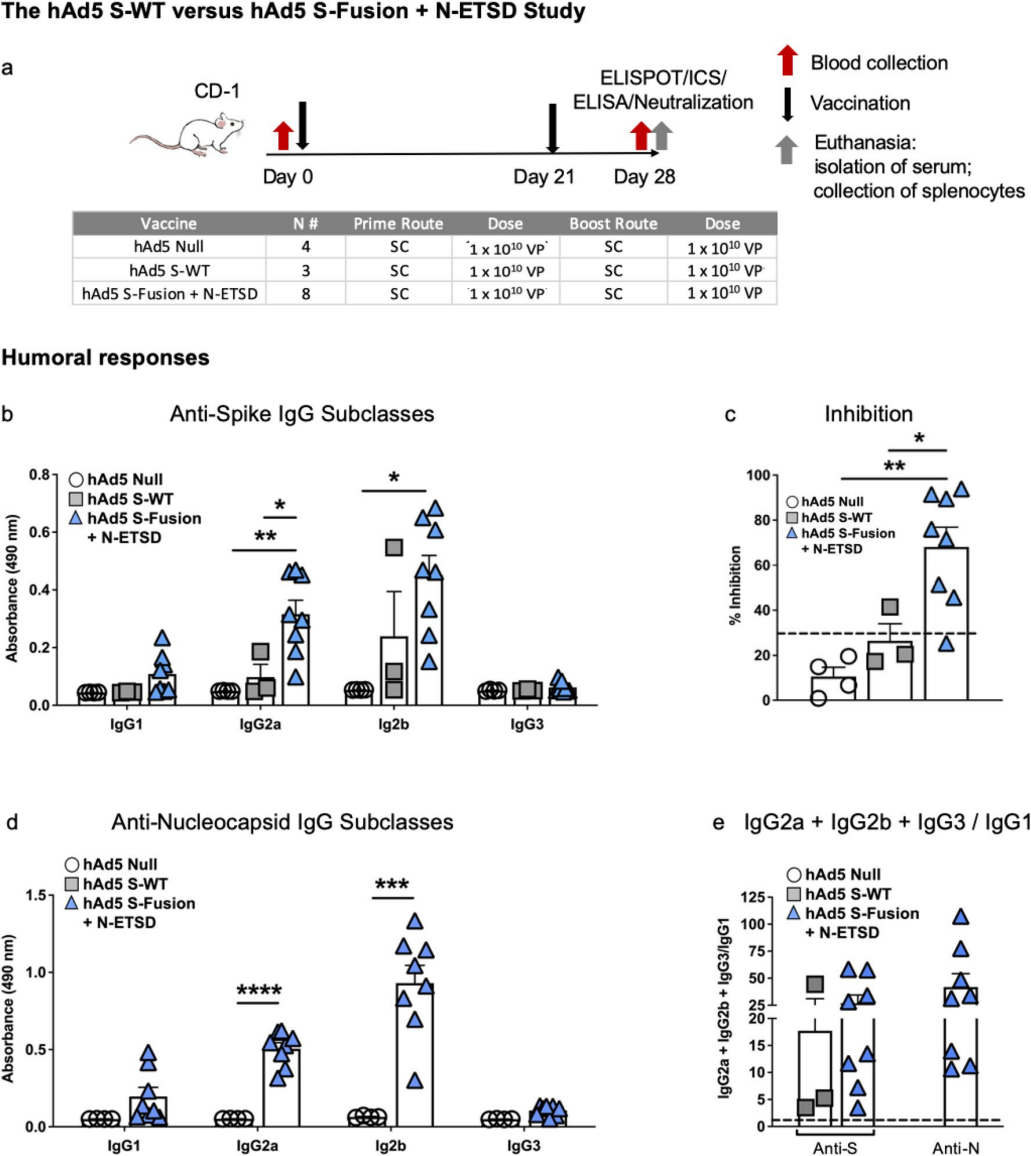
*Humoral responses to S are higher for S-Fusion as compared to S-WT*. (**a**) CD-1 mice received hAd5 Null (n = 4), hAd5 S-WT (n = 3), or the hAd5 S-Fusion + N-ETSD vaccine (n = 8) on Day 0 and Day 21 by subcutaneous (SC) injection and were euthanized for tissue collection on Day 28. (**b**) Anti-S antibody levels in sera by subclass are shown (dilution 1:30) and are higher for S-Fusion + N-ETSD than S-WT. (**c**) Greater inhibition of ACE2: S RBD binding in the surrogate virus neutralization assay was also seen for S-Fusion + N-ETSD as compared to S-WT. Inhibition of 30% (dashed line) or greater is associated with live virus neutralization. (**d**) Anti-nucleocapsid (N) antibody levels (dilution 1:90) by subclass are shown for S-Fusion + N-ETSD only. (**e**) The IgG2a + IgG2b + IgG3/IgG1 ratio calculated using IgG ng equivalents reveals the T helper cell 1 (Th1) bias (> 1, dashed line) for all antibody responses. Statistics performed using one-way Analysis of Variance (ANOVA) with Dunnett’s post-hoc comparison of the S-Fusion + N-ETSD group to S-WT or Null, where *p < 0.05, **p ≤ 0.01, ***p ≤ 0.001, and ****p ≤ 0.0001. Data graphed as the mean with SEM.

For comparison of humoral and T-cell responses to hAd5 S-WT and hAd5 S-Fusion + N-ETSD, CD-1 female mice were inoculated with 1 × 10^10^ viral particles (VP) of hAd5 Null (n = 4), hAd5 S-WT (n = 3) or hAd5 S-Fusion + N-ETSD (n = 8) by subcutaneous (SC) injection on Days 0 and 21. Mice were euthanized and tissue collected for analysis on Day 28 (Fig. 2a).

Generation of IgG2a and 2b anti-S antibodies was significantly greater for mice that received the hAd5 S-Fusion + N-ETSD vaccine as compared to those that received hAd5 S-WT (Fig. 2b). Sera from mice that received the hAd5 S-Fusion + N-ETSD vaccine showed higher inhibition of rACE2 binding to S RBD in the Genscript cPass surrogate assay^47^ than sera from hAd5 S-WT inoculated mice (Fig. 2c). Inhibition of binding in this assay suggests the presence of anti-S RBD neutralizing antibodies and inhibition of 30% or greater is correlated with neutralization of live SARS-CoV-2 virus.

Only mice receiving hAd5 S-Fusion + N-ETSD vaccination generated anti-N antibodies (Fig. 2d), as expected. In hAd5 S-Fusion + N-ETSD mice, anti-N antibody levels were higher than anti-S antibody levels, given the dilution factor in the anti-S ELISA was 1:30 and was 1:90 for the anti-N ELISA.

All humoral responses showed a T helper cell 1 (Th1) bias (Fig. 2e), based on determination of the IgG2a + IgG2b + IgG3 / IgG1 ratio (using ng equivalent values).

### hAd5 S-Fusion + N-ETSD elicits greater T-cell activation than hAd5 S-WT, CD4 + T cells show greater responses to N, and CD8 + T cells show greater responses to S

**Figure 3:**
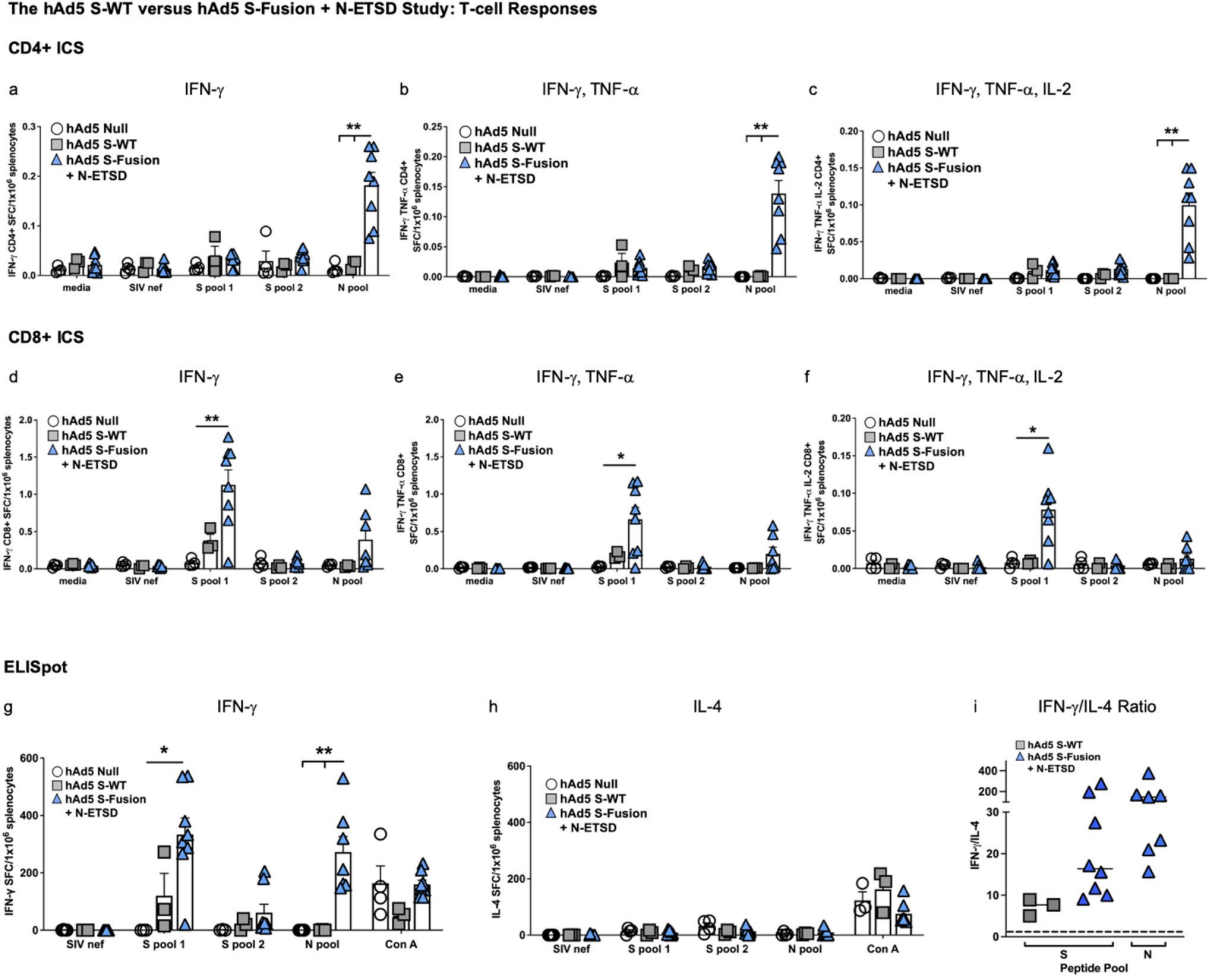
*T-cell responses to S are greater for hAd5 S-Fusion* + *N-ETSD as compared to hAd5 S-WT and CD4* + *T cells were more responsive to N*. Intracellular cytokine staining (ICS) of interferon-γ (IFN-γ), IFN-γ/tumor necrosis factor α (TNF-α), and IFN-γ/TNF-α/interleukin-2 (IL-2) in response to S peptide pools 1 and 2 as well as the N peptide pool is shown for (**a**–**c**) CD4 + and (**d**–**f**) CD8β + T lymphocytes. CD4 + T cells were more responsive to N peptides and CD8 + T cells to S peptide pool 1 that contains the S RBD as compared to S peptide pool 2 or the N pool. Media and SIV nef are negative controls. ELISpot detection of (**g**) IFN-γ secretion by T-lymphocytes also reveals responses to S peptide pool 1 and the N pool. (**h**) Interleukin-4 (IL-4) secretion was very low. SIV nef is a negative control and Con A a positive control. (**i**) The IFN-γ/IL-4 ratios of > 1 (dashed line) reflects the Th1 bias of all T-cell responses. hAd5 Null n = 4, hAd5 S-WT n = 3, and hAd5 S-Fusion + N-ETSD n = 8. Statistical analyses performed using one-way ANOVA with Dunnett’s post-hoc comparison of the S-Fusion + N-ETSD group to the S-WT or Null groups, where *p < 0.05 and **p ≤ 0.01. Data graphed as the mean with SEM.

For mice in the hAd5 S-WT versus hAd5 S-Fusion + N-ETSD study using both SC prime and boost vaccination, Intracellular Cytokine Staining (ICS) revealed that CD4 + T cells were more responsive to the N peptide pool than either S peptide pools 1 or 2 (Fig. 3a–c). Responses to N peptides were only seen for hAd5 S-Fusion + N-ETSD vaccinated mice, as expected.

Conversely, the S1 peptide pool (containing S RBD) elicited higher interferon-γ (IFN-γ), IFN-γ/Tumor necrosis factor-α (TNF-α), and IFN-γ/TNF-α/interleukin-2 (IL-2) production in CD8 + T-lymphocytes than the N peptide pool (Fig. 3d–f). All CD8 + T-cell responses were higher for hAd5 S-Fusion + N-ETSD mice as compared to the mice receiving hAd5 S-WT.

In ELISpot analysis, IFN-γ secretion by T cells from hAd5 S-Fusion + N-ETSD mice in response to S peptide pool 1 and the N peptide pool was similar (Fig. 3g). IFN-γ secretion by T cells from hAd5 S-WT mice was significantly lower. T-cell secretion of interleukin-4 (IL-4) was very low in response to all peptide pools for both vaccinated groups (Fig. 3h), therefore T-cell responses, like humoral responses, showed a Th1 bias with the IFN-γ/IL-4 ratio being > 1 in all mice (Fig. 3i).

### The hAd5 S-Fusion + N-ETSD SC versus IN prime with SC or IN boost study

#### IN prime with an IN boost vaccination with hAd5 S-Fusion + N-ETSD elicits humoral responses as good or better than those with SC prime with either SC or IN boost

**Figure 4:**
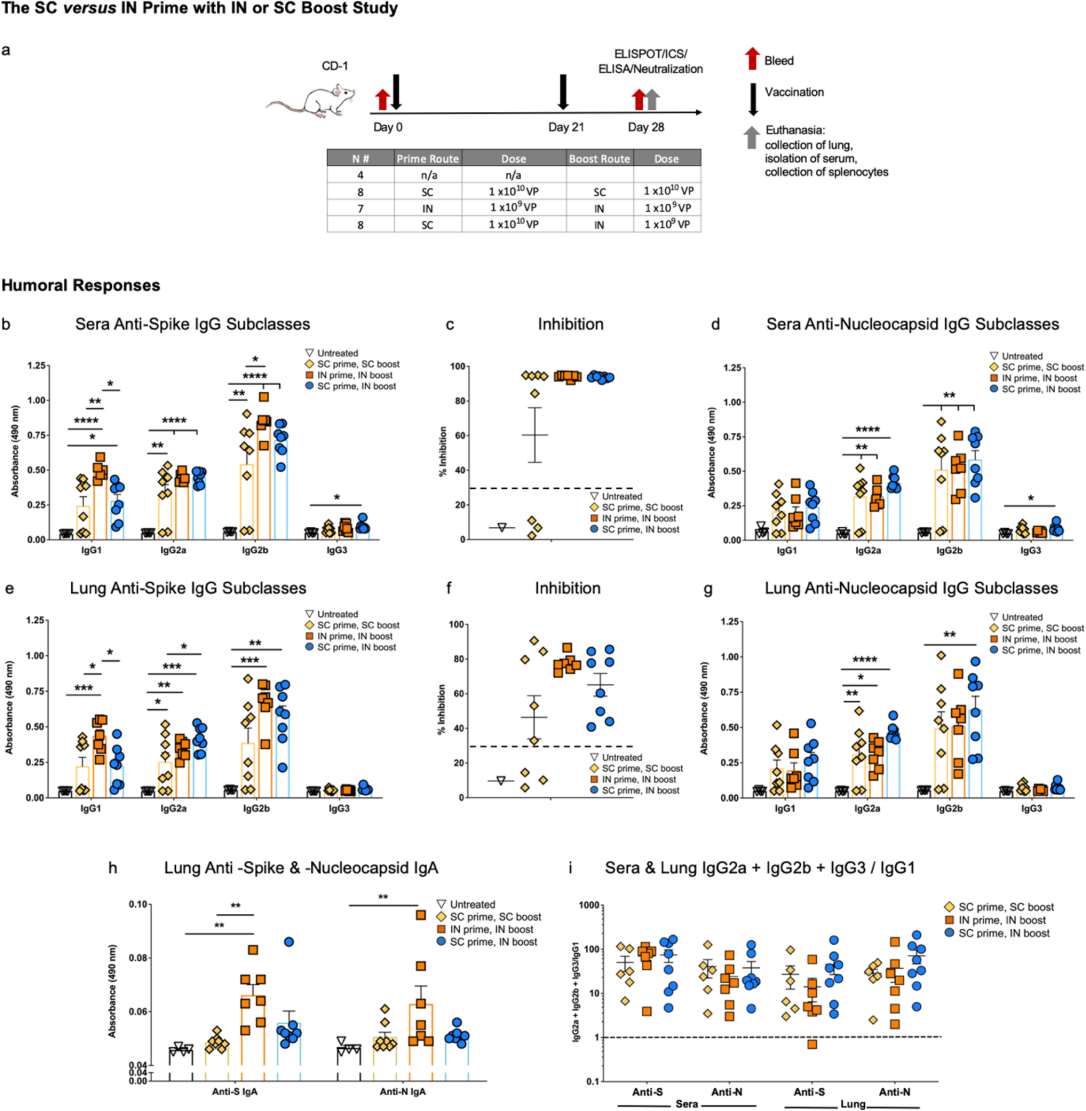
*Humoral responses in the SC versus IN prime with SC or IN boost study*. (**a**) CD-1 mice were untreated (n = 4) or received an SC prime, SC boost (n = 8); IN prime, IN boost (n = 7); or SC prime, IN boost (n = 8). (**b**) Sera anti-S antibodies by subclass (dilution 1:30) are shown as well as (**c**) percent inhibition in the surrogate neutralization assay where inhibition of 30% (dashed line) or greater is correlated with neutralization of live virus. (**d**) Sera anti-N IgG by subclass (dilution 1:270). (**e**) Lung homogenate anti-S IgG by subclass (dilution 1:30), (**f**) inhibition in the surrogate assay, and (**g**) anti-N IgG by subclass (dilution 1:30). (**h**) Lung homogenate anti-S and anti-N IgA. (**i**) The IgG2a + IgG2b + IgG3/IgG1 ratio for sera and lung anti-S and anti-N antibodies where values greater than 1 (dashed line) indicate Th1 bias. The ratio is not represented for mice with very low antibody production. Statistical analyses performed using One-way ANOVA with Tukey’s post-hoc analysis comparing each group to every other group where *p < 0.05; **p ≤ 0.01; ***p ≤ 0.001; and ****p ≤ 0.0001. Data graphed as the mean with SEM.

After establishing that humoral and T-cell responses to the hAd5 S-Fusion + N-ETSD vaccine were greater than those to hAd5 S-WT, we sought to elucidate the potential for the dual antigen vaccine to enhance and broaden immune responses by generation of mucosal immunity. In this study, prime dosing by either SC or IN routes followed by either an SC or IN boost were compared, as shown in Fig. 4a. There were 4 groups of CD-1 mice: untreated, SC prime followed by SC boost (SC > SC), IN prime followed by IN boost (IN > IN), and SC prime followed by IN boost (SC > IN). SC doses were administered at 1 × 10^10^ VP and IN doses were administered at 1 × 10^9^ VP. The untreated group was n = 4, SC > SC and SC > IN were n = 8 and IN > IN n = 7. Mice received the priming doses on Day 0 and boosting doses on Day 21. All mice were euthanized on Day 28 and tissue including blood for serum, spleens for T cells, and lung tissue collected for analyses.

Mice in all vaccinated groups produced anti-S IgG and overall, levels were the highest in sera from IN > IN group mice (Fig. 4b). Sera were highly neutralizing as reflected by inhibition in the surrogate virus neutralization assay in all but 3 mice in the SC > SC group (Fig. 4c). Anti-N IgG was also detected in sera from all vaccinated mice, with the levels being very similar for vaccinated groups (Fig. 4d).

Anti-S IgG was also detected in lung homogenate of all vaccinated mice and was higher overall for the IN > IN group (Fig. 4e). Lung homogenate from all IN > IN group mice showed high inhibition in the surrogate neutralization assay, whereas homogenate from 3 mice in the SC > SC boost group did not surpass the 30% level of inhibition that is associated with viral neutralization (Fig. 4f). In lung homogenate, anti-N IgG showed a trend to be higher in the SC > IN group (Fig. 4g). Not unexpectedly, both anti-S and anti-N IgA levels in lung homogenate were highest in the IN > IN boost group (Fig. 4h). Furthermore, anti-S and anti-N IgG subclass analysis in both sera and lung showed Th1 bias for all vaccinated groups (Fig. 4i).

#### Both CD4 + and CD8 + T-cell responses were greater to N than S, and higher with SC delivery

**Figure 5:**
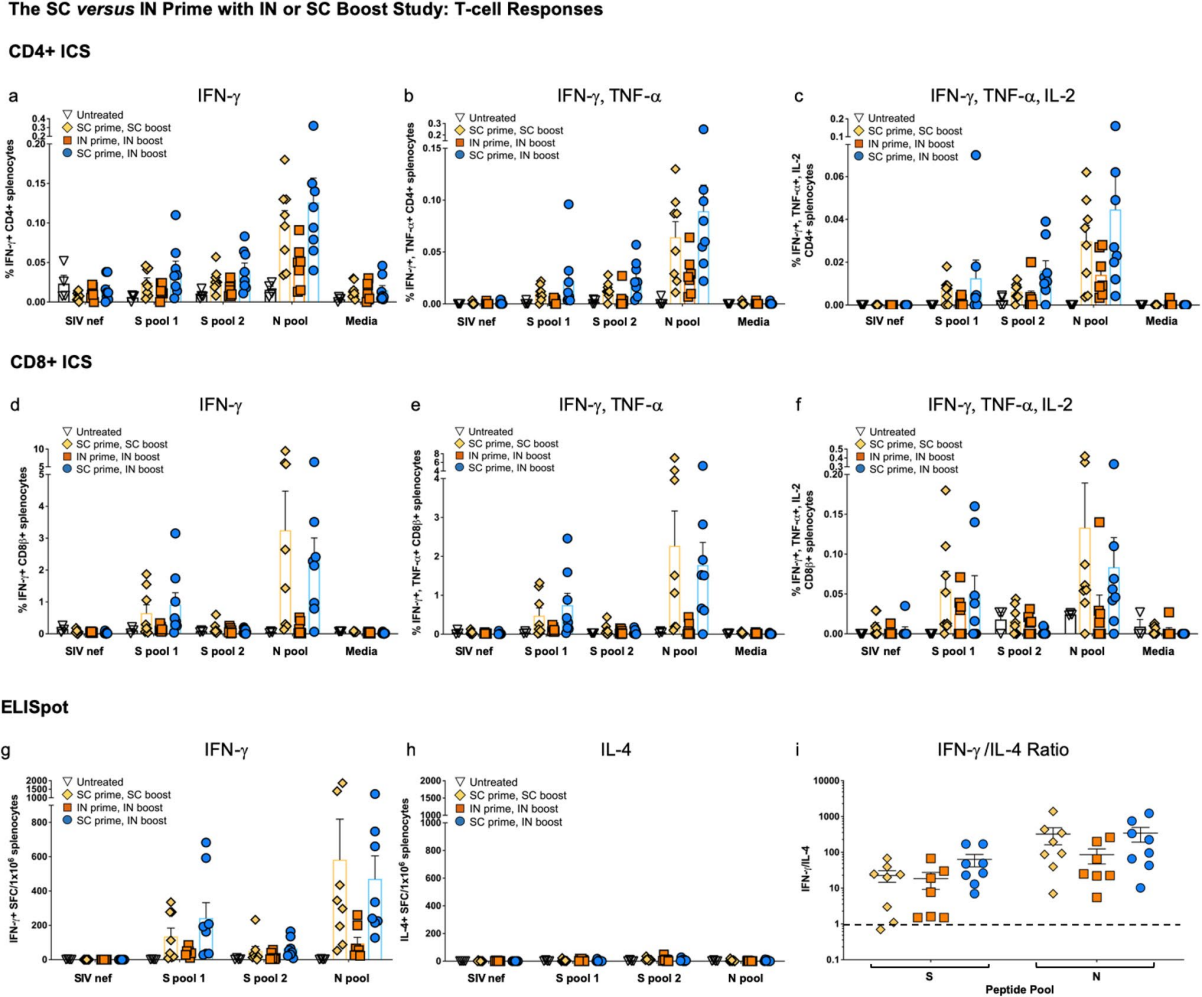
*Both CD4* + *and CD8* + *T cells respond to nucleocapsid peptides in the SC versus IN prime study with SC or IN boost*. ICS of IFN-γ; IFN-γ/TNF-α; and IFN-γ/TNF-α/IL-2 in response to S peptide pools 1 and 2 as well as the N peptide pool for CD4 + (**a–c**) and CD8 + (**d**–**f**) T cells is shown. Some outliers by the Grubb’s test were removed. SIV nef and media are negative controls. ELISpot for (**g**) IFN-γ and (**h**) IL-4 secretion in response to the peptide pools is shown. SIV nef is a negative control. (**i**) The IFN-γ/IL-4 ratios of  > 1 (dashed line) show Th1 bias. Statistical analyses performed using One-way ANOVA with Dunnett’s post-hoc comparison of each treatment group to untreated for each peptide pool was performed but did not reveal statistically significant differences due to individual variation among mice. For untreated n = 4, SC > SC n = 8; IN > IN n = 7, and SC > IN boost n = 8. Data graphed as the mean with SEM.

ICS of IFN-γ (Fig. 5a,d); IFN-γ, TNF-α (Fig. 5b,e), and IFN-γ, TNF-α, interleukin-2 (IL-2) (Fig. 5c,f) showed the highest mean values for the SC > SC boost and SC > IN vaccinated groups with responses to the N peptide pool trending higher for both CD4 + and CD8 + T cells. This was somewhat in contrast with the findings of the first SC > SC study, where CD8 + T-cell responses were greater to the S1 peptide pool (Fig. 3d–f), however, variation is expected in outbred CD-1 mice and robust CD8 + responses to both S and N were detected in SC > SC mice from each study. While the differences were not statistically significant due to variation among individual mice, overall the IN > IN boost group had a reduced population of CD8 + cells capable of accumulating cytokines in response to S and N peptide stimulation (Fig. 5d–f).

ELISpot findings were similar, with higher responses seen for the SC > SC and SC > IN groups when compared to the IN > IN group (Fig. 5g). The highest responses were found to be specific to the N peptide pool. Interleukin-4 (IL-4) secretion in ELISpot was very low for all groups (Fig. 5h), therefore the IFN-γ/IL-4 ratios were above 1 for almost all vaccinated mice in response to both S and N peptide pools (Fig. 5i), indicating Th1 bias.

The findings in this study suggest an important contribution of SC delivery to T cell responses.

### The combined SC + IN prime with or without SC or IN boost study

#### Prime-only delivery by combined SC and IN dosing elicits humoral responses that are as good or better than those with a boost

**Figure 6:**
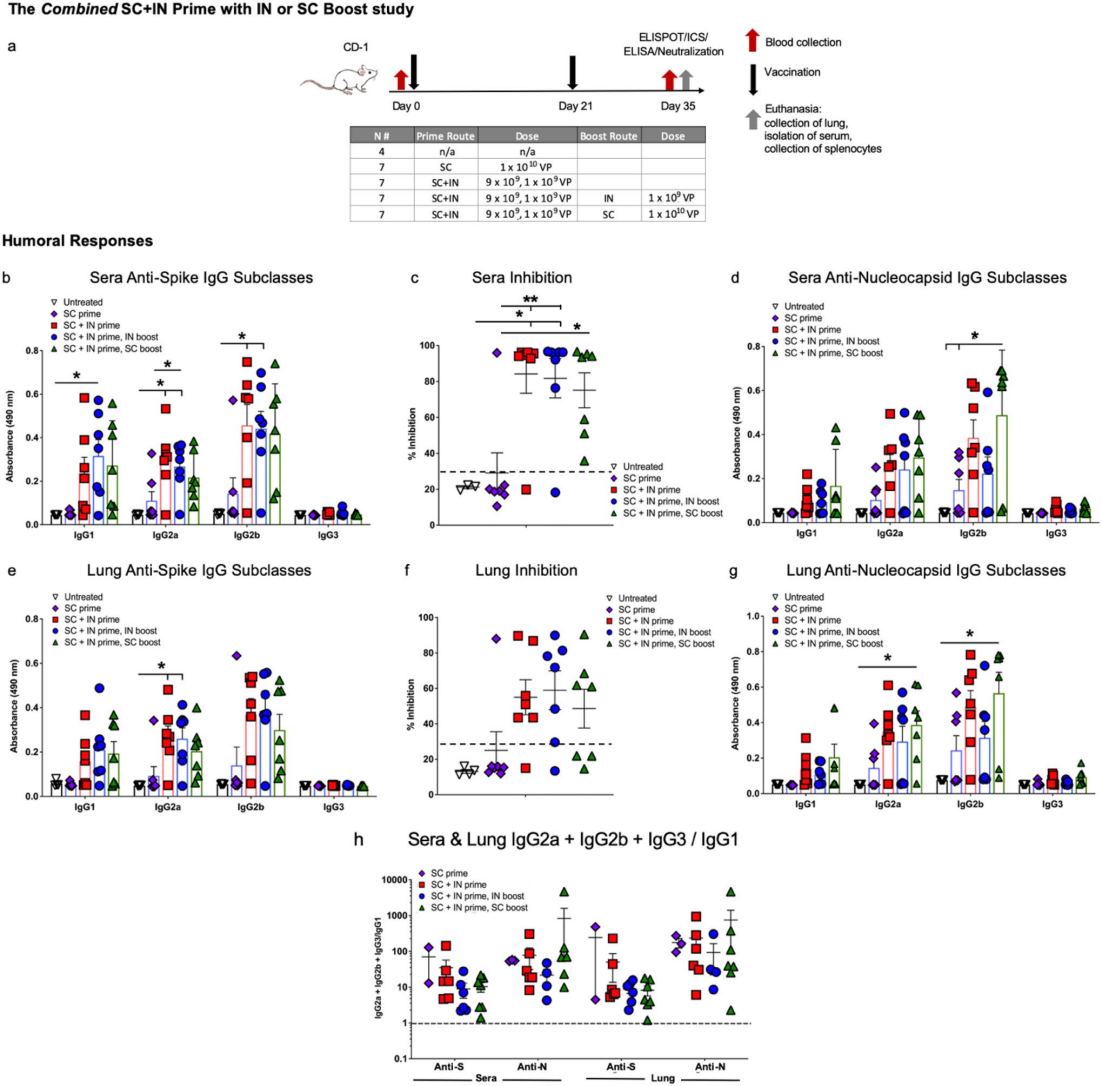
*Subcutaneous (SC) plus intranasal (IN) prime without boost elicits Th1 biased neutralizing anti-S and anti-N antibodies.* (**a**) The study design is shown with groups for SC prime only, SC + IN prime only, and SC + IN prime with either an SC or IN boost, all n = 7. There was an untreated control group of n = 4. Prime dosing was on Day 0, boosts on Day 21, and euthanasia on Day 35. Shown are sera (**b**) anti-spike (S) antibodies by subclass (dilution 1:30); (**c**) percent inhibition in the surrogate neutralization assay with sera where > 30% (dashed line) is correlated with neutralization of virus; and (**d**) anti-nucleocapsid (N) antibodies (dilution 1:270). Lung homogenate (**e**) anti-S antibodies; (f) neutralization (30% is dashed line); and (**g**) anti-N antibodies (dilution 1:30 for anti-S and -N). (**h**) The IgG1a + IgG2b + IgG3/IgG1 ratios for anti-S and anti-N antibodies are shown for sera and lung; values > 1 (dashed line) indicate Th1 bias. The ratio is not represented for mice with very low antibody production. Statistical analyses performed using One-way ANOVA with Tukey’s post-hoc analysis comparing groups where *p < 0.05 and **p ≤ 0.01. Data graphed as the mean with SEM.

To leverage both the humoral responses effectively elicited by IN delivery with the T-cell responses that were greater with SC delivery, we then tested prime delivery by a combination of the SC and IN routes, with either IN or SC boosts. This study design is shown in Fig. 6a. There were 5 groups of CD-1 mice: untreated, an SC prime at 1 × 10^10^ viral particles (VP) without boost (SC > no boost), a combined 9 × 10^9^ VP SC plus 1 × 10^9^ VP IN prime (SC + IN) without boost (SC + IN > no boost), a combined SC + IN prime with 1 × 10^9^ VP IN boost (SC + IN > IN), and a combined SC + IN prime with a 1 × 10^10^ VP SC boost (SC + IN > SC). The untreated group was n = 4 and all vaccinated groups were n = 7. Mice received the prime on Day 0 and in appropriate groups, the boost on Day 21. All mice were euthanized on Day 35 and tissue including blood for serum, spleens for T cells, and lung tissue collected for analyses. Note this euthanasia day is one week later than the two studies described above (Figs. 2a, 4a), which was a change meant to better characterize humoral responses at a time point at which we expected cell-mediated responses to remain high based on our prior work with this vaccine platform.

The combined SC + IN > no boost regimen was just as effective in eliciting neutralizing anti-S IgG and anti-N IgG antibody production in both sera (Fig. 6b–d) and lung (Fig. 6e–g) as either the SC + IN > IN or SC + IN > SC regimens. SC > no boost gave significantly lower humoral responses (Fig. 6b–g). All humoral responses showed a Th1 bias (Fig. 6h) based on the ratio of IgG2a, IgG2b, and IgG3 to IgG1 antibody subclasses.

#### SC plus IN prime alone without a boost elicits CD4 + T cell responses to N and CD8 + T-cell responses to S

**Figure 7:**
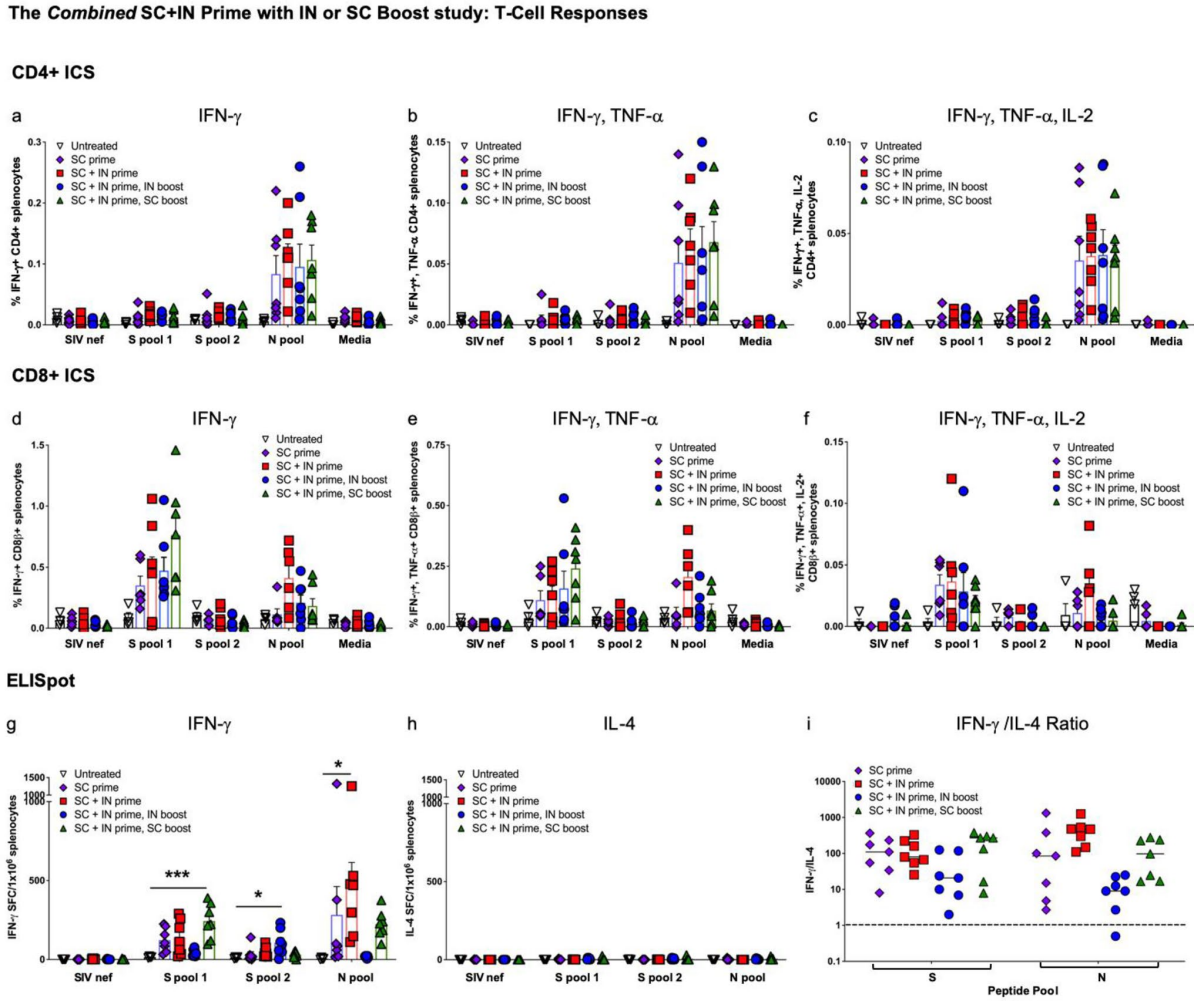
*CD4* + *T cells respond to nucleocapsid (N) and CD8* + *T cells to spike in the combined SC plus IN prime study with SC or IN boost*. ICS of IFN-γ; IFN-γ/TNF-α; and IFN-γ/TNF-α/IL-2 in response to S peptide pools 1 and 2 as well as the N peptide pool for CD4 + (**a**–**c**) and CD8 + (**d**–**f**) T lymphocytes is shown. Some outliers by the Grubb’s test were removed. SIV nef and media are negative controls. ELISpot for (**g**) IFN-γ and (**h**) interleukin-4 (IL-4) secretion in response to the peptide pools I shown. SIV nef is a negative control. (**i**) The IFN-γ/IL-4 ratios where values > 1 (dashed line) indicate Th1 bias. Statistical analyses performed using One-way ANOVA with Dunnett’s post-hoc comparison of each group to untreated where *p < 0.05, **p ≤ 0.01, and ***p ≤ 0.001. Untreated n = 4 and all vaccinated groups n = 7 (unless removed as an outlier). Data graphed as the mean with SEM.

Similar to the findings in the first study, ICS shows the N peptide pool stimulated cytokine production by CD4 + T lymphocytes from all vaccinated mice (Fig. 7a–c), but CD8 + T cells from vaccinated mice responded to S peptide pool 1 which contains the S RBD (Fig. 7d–f). The differences between vaccinated groups were not significant due to variability among mice, with SC + IN > no boost vaccinated mice having T-cell responses that were similar to those seen with mice that did receive boosts.

In ELISpot, the highest IFN-γ secretion in response to peptide pools differed by both peptide pool and vaccination regimen. As compared to the negative control (SIV nef), T-cell IFN-γ secretion was significantly greater for the combined SC + IN > SC group in response to the S1 peptide pool; greater for the SC + IN > IN group to the S2 peptide pool; and greater for the SC + IN > no boost group to the N peptide pool (Fig. 7g).

IL-4 secretion was very low (Fig. 7h), therefore the IFN-γ/IL-4 ratio was above 1 for all vaccinated mice with only one exception, reflecting Th1 bias of T-cell responses (Fig. 7i).

## Discussion

Our hAd5 S-Fusion + N-ETSD vaccine was designed to overcome the risks of an S-only vaccine and elicit both T-cell immunity and neutralizing antibodies, leveraging the vital role T cells play in generating long-lasting antibody responses and in directly killing infected cells. The CD4 + and CD8 + T cell responses induced by this vaccine are multifunctional, and induction of such multifunctional T cells by vaccines is correlated with better protection against infection^48^. We postulated that enhanced CD4 + T-cell responses and Th1 predominance resulting from expression of an S antigen optimized for surface display and an N antigen optimized for endosomal/lysosomal subcellular compartment localization^1^ and thus MHC I and II presentation, led to increased dendritic cell presentation, cross-presentation, B cell activation, and ultimately high neutralization capability as determined in the surrogate assay.

It is well established that the contemporaneous MHC I and MHC II presentation of an antigen by the antigen presenting cell activates CD4 + and CD8 + T cells simultaneously and is optimal for the generation of memory B and T cells. A key finding of our construct is that N-ETSD elicits a CD4 + T-cell response, a necessity for induction of memory T cells and helper cells for B cell antibody production. Others have also reported on the importance of lysosomal localization for eliciting the strongest T-cell IFN-γ and CTL responses, compared to natural N^49,50^.

The T-cell responses to the S and N antigens expressed by hAd5 S-Fusion + N-ETSD were polycytokine, including IFN-γ, TNF-α, and IL-2, consistent with successful antimicrobial immunity in bacterial and viral infections^51–55^. Post-vaccination polycytokine T-cell responses have been shown to correlate with vaccine efficacy, including those with a viral vector^48^. Of relevance here, the polycytokine T-cell responses to SARS-CoV-2 N protein are consistent with those observed in recovered COVID-19 patients^27^, supporting the rationale that the dual antigen hAd5 S-Fusion + N-ETSD vaccine-induced immune responses against both SARS-CoV-2 and N antigens may provide vaccinated subjects with greater protections against SARS-CoV-2, including variants.

In future studies utilizing animal models of SARS-CoV-2 challenge, we hope to elucidate the role of S and N individually and in combination, as well as the relative importance of humoral and cell-mediated immune responses, in providing protection against SARS-CoV-2 infection.

The key finding here that prime-only vaccination delivered by combination SC and IN dosing results in broad humoral and systemic T-cell responses, along with the potential for enhanced mucosal immunity, supports the clinical testing of the hAd5 S-Fusion + N-ETSD vaccine by these simultaneous routes of immunization. The vaccine has currently completed Phase 1 testing as an SC prime and SC boost. Oral boost formulations that have shown efficacy in the ability to elicit immune responses that conferred complete protection against high-titer SARS-CoV-2 challenge in our pre-clinical studies in non-human primates^56^ will soon also be tested in the clinic. To our knowledge, our vaccine is currently the only one available in SC, oral^57^, and IN formulations that offer expanded possibilities for efficient, feasible delivery across the globe.

## Methods

### The hAd5 [E1-, E2b-, E3-] platform and constructs

**Figure 8:**
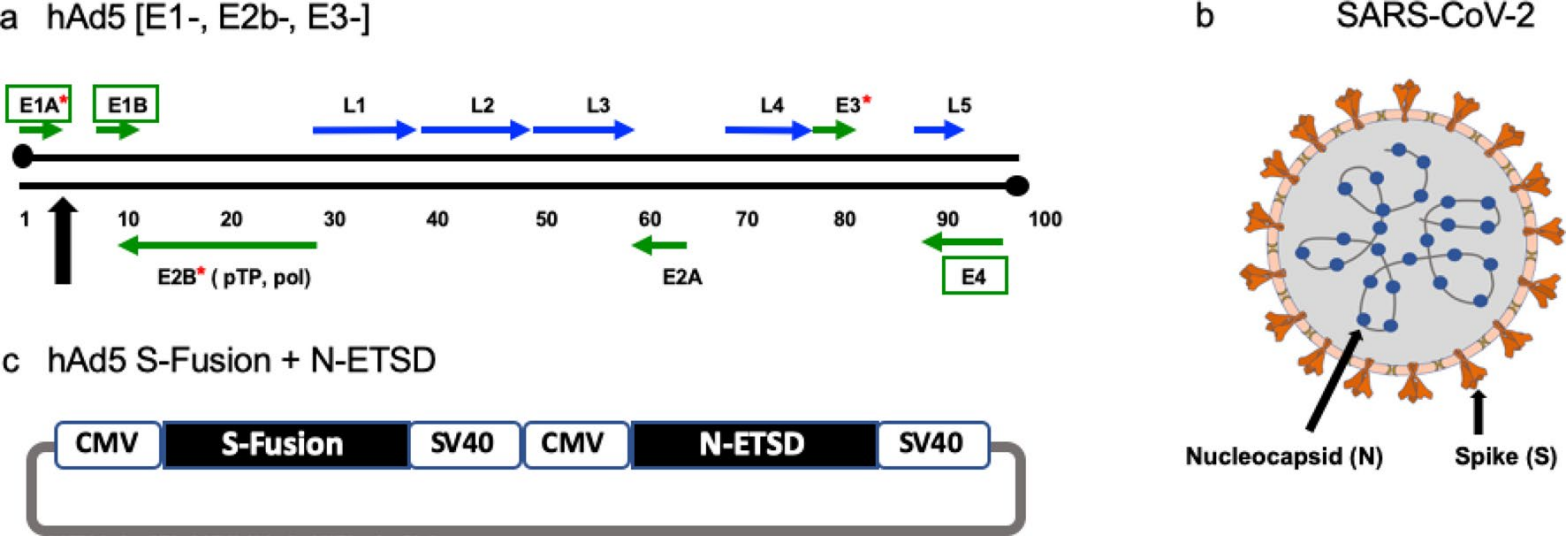
*The SARS-CoV-2 virus, the hAd5 [E1-, E2b-, E3-] vector and the dual antigen hAd5 S-Fusion* + *N-ETSD vaccine.* (**a**) The second-generation human adenovirus serotype 5 (hAd5) vector used has the E1, E2b, and E3 regions deleted. Sequences for the vaccine antigen cargo are inserted at the black arrow. (**b**) The spike (S) glycoprotein is displayed as a trimer on the surface of SARS-CoV-2 and the nucleocapsid (N) protein is found in the virus interior, associated with the viral RNA. (**c**) The vaccine antigens are under control of the cytomegalovirus (CMV) promoter and sequences end with SV40 poly-A.

For studies here, the next generation hAd5 [E1-, E2b-, E3-] vector was used (Fig. 8a) to create viral vaccine candidate constructs. This hAd5 [E1-, E2b-, E3-] vector is primarily distinguished from other first-generation [E1-, E3-] recombinant Ad5 platforms^58,59^ by having additional deletions in the early gene 2b (E2b) region that remove the expression of the viral DNA polymerase (pol) and in pre terminal protein (pTP) genes, and its propagation in the E.C7 human cell line^2,3,5,60^.

The hAd5 S-Fusion + N-ETSD vaccine we utilized comprises the hAd5 [E1-, E2b-, E3-] vector with a wild type spike (S) sequence [accession number YP009724390] modified with a proprietary linker peptide sequence as well as a wild type nucleocapsid (N) sequence [accession number YP009724397] with a an Enhanced T-cell Stimulation Domain (ETSD) signal sequence to direct translated N to the endosomal/lysosomal pathway^1^. The SARS-CoV-2 S protein is found on the viral surface^15^ and the N protein is found in the interior of the virus^30,61^ (Fig. 8b).

The powerful cytomegalovirus (CMV) promoter^62^ drives expression in the hAd5 construct (Fig. 8c).

### Infection (transduction) of HEK-293T cells and flow cytometry for anti-RBD antibody binding

Relative levels of cell-surface expression of S RBD after transduction of human embryonic kidney (HEK) 293T cells with various S-expressing hAd5 vaccines were determined by flow cytometry. HEK 293T cells (2.5 × 10^5^ cells/well in 24 well plates) were grown in DMEM containing 10% FBS and PSA (100 units/mL penicillin, 100 μg/mL streptomycin, 0.25 μg/mL Amphotericin B) at 37 °C. Cells were either untransduced or transduced with hAd5 S-WT, S-WT + N-ETSD, S-Fusion, or S-Fusion + N-ETSD viral particles at a multiplicity of infection (MOI) of 10. For detection of S RBD 24 hurs after transduction, cells were transferred by gentle pipetting into medium and labeled with an anti-RBD monoclonal antibody (clone D003 Sino Biological) and F(ab’)2-Goat anti-Human IgG-Fc secondary antibody conjugated with R-phycoerythrin (Thermofisher). Labeled cells were acquired using a Thermo-Fisher Attune NxT flow cytometer and analyzed using Flowjo Software.

### Transfection of HEK 293T cells with hAd5 constructs and flow cytometric analysis of recombinant ACE2 binding

For recombinant ACE2 binding experiments, HEK 293T cells were transfected with hAd5 construct DNA. The constructs tested were: S-WT, S-Fusion, and S-Fusion + N-ETSD. HEK 293T cells (2.5 × 10^5^ cells/well in 24 well plates) were grown in DMEM (Gibco Cat# 11995-065) with 10% FBS and 1X PSA (100 units/mL penicillin, 100 μg/mL streptomycin, 0.25 ug/mL Amphotericin B) at 37 °C. Cells were transfected with 0.5 μg of hAd5 plasmid DNA using a JetPrime transfection reagent (Polyplus Catalog # 89129-924) according to the manufacturer’s instructions. Cells were harvested 48 h post transfection by gently pipetting cells into medium and labeled with recombinant ACE2-Fc. Recombinant ACE2-IgG1Fc protein was produced using Maxcyte transfection in CHO-S cells that were cultured for 14 days. ACE2-IgG1Fc was then purified using a MabSelect SuRe affinity column on AKTA Explorer. Purified ACE2-IgG1Fc was dialyzed into 10 mM HEPES, pH7.4, 150 mM NaCl and concentrated to 2.6 mg/mL. For binding studies, the ACE2-IgG1Fc was used at a concentration of 1 μg/mL for binding. Cells were incubated with ACE2-Fc for 20 min and, after a washing step, were then labeled with a PE conjugated F(ab’)2-goat anti-human IgG Fc secondary antibody at a 1:100 dilution, incubated for 20 min, washed and acquired on flow cytometer. Histograms are based on normalized mode (NM) of cell count—count of cells positive for signal in PE channel.

### Murine immunization and blood/tissue collection

All in vivo experiments described were carried out in strict accordance with good animal practice according to NIH recommendations. All procedures for animal use were approved by the IACUC Committee at Omeros, Inc. (Seattle, WA, USA) and under an approved protocol.

CD-1 female mice (Charles River Laboratories) 6–8 weeks of age were used for immunological studies performed at the vivarium facilities of Omeros Inc. (Seattle, WA). Mice were administered subcutaneous (SC) injections at the indicated doses in 50 µL ARM buffer (20 mM Tris pH 8.0, 25 mM NaCl, with 2.5% glycerol) or intranasal (IN) injections at the indicated doses in 10 µL ARM buffer (5 µL per nostril) while under isoflurane anesthesia. On the final day of each study, blood was collected via the submandibular vein from isoflurane-anesthetized mice for isolation of sera using a microtainer tube and then mice were euthanized for collection of spleen and lungs.

Spleens were removed from each mouse and placed in 5 mL of sterile media (RPMI/HEPES/Pen/Strep/10% FBS). Splenocytes were isolated within 2 h of collection and used fresh or frozen for later analysis.

Lungs were removed from each mouse, dissected in half and then immediately snap frozen on dry ice. Lung homogenates were generated by thawing one frozen lung half and homogenizing in 150 μL sterile PBS using a Fisher Scientific pestle drill. Homogenates were centrifuged at 13,000 rpm for 3 min and supernatants were utilized in ELISA and GenScript cPass surrogate neutralization assays.

### Intracellular cytokine stimulation (ICS)

ICS assays were performed using 10^6^ live splenocytes per well in 96-well U-bottom plates. Splenocytes in RPMI media supplemented with 10% FBS were stimulated by the addition of pools of overlapping peptide for S or N antigens at 2 μg/mL/peptide for 6 h at 37 °C in 5% CO_2_, with protein transport inhibitor, GolgiStop (BD) added two hours after initiation of incubation. The S peptide pool (JPT: Cat #PM-WCPV-S-1) is a total of 315 spike peptides split into two pools comprised of 158 and 157 peptides each. The N peptide pool (JPT; Cat # PM-WCPV-NCAP-1) was also used to stimulate cells. A SIV-Nef peptide pool (BEI Resources) was used as an off-target negative control. Stimulated splenocytes were then stained for a fixable cell viability stain followed by the lymphocyte surface markers CD8β and CD4, fixed with CytoFix (BD), permeabilized, and stained for intracellular accumulation of IFN-γ, TNF-α and IL-2 (in studies 2 and 3). Fluorescent-conjugated antibodies against mouse CD8β antibody (clone H35-17.2, ThermoFisher), CD4 (clone RM4-5, BD), IFN-γ (clone XMG1.2, BD), TNF-α (clone MP6-XT22, BD) and IL-2 (clone JES6-5H4; BD), and staining was performed in the presence of unlabeled anti-CD16/CD32 antibody (clone 2.4G2; BD). Flow cytometry was performed using a Beckman-Coulter Cytoflex S flow cytometer and analyzed using Flowjo Software.

### ELISpot assay

ELISpot assays were used to detect cytokines secreted by splenocytes from inoculated mice. Fresh splenocytes were used on the same day, as were cryopreserved splenocytes containing lymphocytes. The cells (2–4 × 10^5^ cells per well of a 96-well plate) were added to the ELISpot plate containing an immobilized primary antibody to either IFN-γ or IL-4 (BD), and were exposed to various stimuli (e.g. control peptides, target peptide pools/proteins) comprising 2 μg/mL peptide pools or 10 μg/mL protein for 36–40 h. After aspiration and washing to remove cells and media, extracellular cytokine was detected by a secondary antibody to cytokine conjugated to biotin (BD). A streptavidin/horseradish peroxidase conjugate was used detect the biotin-conjugated secondary antibody. The number of spots per well, or per 2–4 × 10^5^ cells, was counted using an ELISpot plate reader. Quantification of Th1/Th2 bias was calculated by dividing the IFN-γ spot forming cells (SFC) per million splenocytes with the IL-4 SFC per million splenocytes for each animal.

### ELISA for detection of antibodies

For IgG antibody detection in sera and lung homogenate from inoculated mice, ELISAs specific for spike and nucleocapsid antibodies, as well as for IgG subclass (IgG1, IgG2a, IgG2b, and IgG3) antibodies were used. In addition, for IgA antibody detection in lung homogenate from inoculated mice, ELISAs specific for spike and nucleocapsid antibodies, as well as for IgA was used. A microtiter plate was coated overnight with 100 ng of either purified recombinant SARS-CoV-2 S-FTD (full-length S with fibritin trimerization domain, constructed and purified in-house by ImmunityBio), SARS-CoV-2 S RBD (Sino Biological, Beijing, China; Cat # 401591-V08B1-100) or purified recombinant SARS-CoV-2 nucleocapsid (N) protein (Sino Biological, Beijing, China; Cat # 40588-V08B) in 100 µL of coating buffer (0.05 M Carbonate Buffer, pH 9.6). The wells were washed three times with 250 µL PBS containing 1% Tween 20 (PBST) to remove unbound protein and the plate was blocked for 60 min at room temperature with 250 µL PBST. After blocking, the wells were washed with PBST, 100 μL of either diluted serum or diluted lung homogenate samples were added to wells, and samples incubated for 60 min at room temperature. After incubation, the wells were washed with PBST and 100 μL of a 1/5000 dilution of anti-mouse IgG HRP (GE Health Care; Cat # NA9310V), or anti-mouse IgG_1_ HRP (Sigma; Cat # SAB3701171), or anti-mouse IgG_2a_ HRP (Sigma; Cat # SAB3701178), or anti-mouse IgG_2b_ HRP (Sigma; catalog# SAB3701185), anti-mouse IgG_3_ HRP conjugated antibody (Sigma; Cat # SAB3701192), or anti-mouse IgA HRP conjugated antibody (Sigma; Cat # A4789) was added to wells. For positive controls, a 100 μL of a 1/5000 dilution of rabbit anti-N IgG Ab or 100 μL of a 1/25 dilution of mouse anti-S serum (from mice immunized with purified S antigen in adjuvant) were added to appropriate wells. After incubation at room temperature for 1 h, the wells were washed with PBS-T and incubated with 200 μL o-phenylenediamine-dihydrochloride (OPD substrate (Thermo Scientific Cat # A34006) until appropriate color development. The color reaction was stopped with addition of 50 μL 10% phosphoric acid solution (Fisher Cat # A260-500) in water and the absorbance at 490 nm was determined using a microplate reader (SoftMax Pro, Molecular Devices).

### Calculation of relative ng amounts of antibodies and Th1/Th2 IgG subclass bias

A standard curve of IgG was generated and absorbance values were converted into mass equivalents for both anti-S and anti-N antibodies. Using these values, we were able to calculate that hAd5 S-Fusion + N-ETSD vaccination generated a geometric mean value for S- and N-specific IgG per milliliter of serum. These values were also used to quantify the Th1/Th2 bias for the humoral responses by dividing the sum total of Th1 biased antigen-specific IgG subclasses (IgG2a, IgG2b and IgG3) with the total Th2 skewed IgG3, for each mouse. For mice that lack anti-S and/or anti-N specific IgG responses, Th1/Th2 ratio was not calculated. Conversely, some responses, particularly for anti-N responses in IgG2a and IgG2b (both Th1 biased subclasses), were above the limit of quantification with OD values higher than those observed in the standard curve. These data points were reduced to values within the standard curve, and thus will reflect a lower Th1/Th2 bias than would otherwise be reported.

### GenScript cPass neutralizing antibody detection

The GenScript cPass assay (https://www.genscript.com/cpass-sars-cov-2-neutralization-antibody-detection-Kit.html) for detection of neutralizing antibodies was used according to the manufacturer’s instructions^47^. The kit detects circulating (sera) neutralizing antibodies against SARS-CoV-2 that block the interaction between the S RBD with the ACE2 cell surface receptor. It is suitable for all antibody subclasses and appropriate for use with in animal models without modification.

### Statistical analyses and graph generation

All statistical analyses were performed and graphs used in figures were generated using GraphPad Prism software. Statistical tests for each graph are described in the figure legends.
